# Adipocyte-derived estrogen signaling influences TROP2 expression in ERα-positive breast cancer

**DOI:** 10.3389/fendo.2026.1866290

**Published:** 2026-08-12

**Authors:** Luca Gelsomino, Linda Manna, Vittoria Marchio, Piercarlo Del Console, Giuseppina Augimeri, Cinzia Giordano, Balázs Győrffy, Daniela Bonofiglio, Carmine De Angelis, Stefania Catalano, Ines Barone

**Affiliations:** 1 Department of Pharmacy, Health and Nutritional Sciences, University of Calabria, Cosenza, Italy; 2 Centro Sanitario, University of Calabria, Cosenza, Italy; 3 Clinical Laboratory Unit, Azienda Ospedaliera (AO) “Annunziata”, Cosenza, Italy; 4 Department of Bioinformatics, Semmelweis University, Budapest, Hungary; 5 Institute of Transdisciplinary Discoveries, Medical School, University of Pecs, Pecs, Hungary; 6 Cancer Biomarker Research Group, Institute of Molecular Life Sciences, Hungarian Research Network, Budapest, Hungary; 7 Clinical and Translational Oncology, Scuola Superiore, Naples, Italy; 8 National Cancer Institute, Istituto di Ricovero e Cura a Carattere Scientifico (IRCCS) Fondazione G. Pascale, Naples, Italy

**Keywords:** ADCs, adipocytes, estrogen receptor alpha, obesity, TROP2, breast cancer

## Abstract

**Background:**

Obesity-associated alterations in the tumor microenvironment (TME) modulate breast cancer (BC) progression. In obese individuals, adipocytes, the predominant stromal component of breast tissue, display an altered endocrine and inflammatory profile, leading to increased secretion of hormones, adipokines, and cytokines that influence epithelial cell behavior and contribute to tumor progression and therapeutic resistance. In the metastatic setting, antibody–drug conjugates (ADCs) have expanded the therapeutic landscape of BC. However, resistance remains a clinical challenge, with loss or downregulation of the target antigen representing a key mechanism. Increasing evidence indicates that TME-derived signals regulate therapeutic target expression. Among these, Trophoblast Cell Surface Antigen 2 (TROP2), a clinically validated ADC target, can be modulated by stromal-derived factors Here, we investigated whether adipocytes regulate TROP2 expression and influence the efficacy of TROP2-directed ADCs in BC cells.

**Methods:**

Conditioned media (CM) from differentiated 3T3-L1 adipocytes were applied to ERα-positive (MCF-7, T47D) and ERα-negative (MDA-MB-231) BC cells. TROP2 expression was evaluated by qRT-PCR and immunoblotting. Estradiol levels in adipocyte CM were quantified by ELISA, and ERα signaling was inhibited using tamoxifen and fulvestrant. In silico analyses assessed the relationship between ERα signaling and TACSTD2 expression and evaluated TACSTD2 levels in BC cohorts stratified by body mass index (BMI). Cell viability assays determined responsiveness to Sacituzumab Govitecan and Datopotamab Deruxtecan.

**Results:**

Adipocyte-derived CM significantly reduced TROP2 expression at transcript and protein levels, selectively in ERα-positive BC models. Elevated 17β-estradiol levels were detected in adipocyte CM, and estradiol exposure recapitulated TROP2 downregulation. Pharmacological inhibition of ERα signaling or the blockade of estrogen production abrogated this effect, supporting a role for ERα activation. In silico analyses suggested a functional link between ERα and TROP2. TROP2 expression was reduced in tumors from overweight/obese patients with ERα-positive BC, but not in ERα-negative cases. Functionally, adipocyte CM counteracted the antiproliferative effects of Sacituzumab Govitecan and Datopotamab Deruxtecan in ERα-positive BC cells, an effect that was reversed by ERα inhibition.

**Conclusions:**

These findings identify an estrogen-dependent, adipocyte-driven mechanism that modulates TROP2 expression and reduces responsiveness to TROP2-directed ADCs in ERα-positive BC models. Our data highlight the contribution of obesity-associated adipose tissue dysfunction to variability in targeted therapy response and warrant further investigation in clinically relevant settings.

## 1 Introduction

According to GLOBOCAN 2022, breast cancer remains the most frequently diagnosed malignancy and the leading cause of cancer-related deaths among women worldwide (1). Beyond tumor-intrinsic genetic alterations, increasing evidence indicates that disease progression is strongly influenced by host-related factors and the tumor microenvironment (TME). Among these, obesity has emerged as a major determinant of breast cancer risk and outcomes, particularly in postmenopausal women and in estrogen receptor alpha (ERα)–positive disease. Obesity-associated changes in adipose tissue promote a pro-tumorigenic environment characterized by chronic inflammation, altered adipokine secretion, metabolic dysregulation, and increased local estrogen production through aromatase activity (reviewed in (2–4)).

Beyond their contribution to systemic obesity-associated disturbances, adipocytes represent a major cellular component of the breast tumor microenvironment and actively interact with cancer cells, promoting proliferation, migration, invasion, and metabolic reprogramming across multiple breast cancer models (5–8). Indeed, obese adipocytes are characterized by altered secretion of bioactive molecules that can promote hyperactivation of key cancer cell–intrinsic signaling pathways, including the MAPK and PI3K/Akt cascades. Through the engagement of specific transmembrane receptors, these signals can influence therapeutic responses and sustain malignant progression (3, 4).

The Trophoblast Cell Surface Antigen 2 (TROP2), a transmembrane glycoprotein and cell-surface receptor encoded by the *TACSTD2* gene, has emerged as an attractive molecular candidate for targeted therapeutics in several epithelial malignancies, including breast cancer (9–12). Aberrant upregulation of TROP2 has been documented in several breast cancer subtypes, particularly in triple-negative disease, whereas its expression in normal tissues is typically limited (13). Moreover, TROP2 has been associated with enhanced tumor cell proliferation, invasion, and poor clinical outcome (14–16).

Owing to its cell-surface localization and frequent expression in breast tumors, TROP2 has emerged as a promising target for antibody-based therapies. Antibody-drug conjugate (ADC)-based anti-TROP2 therapies represent a relatively novel class of anticancer agents designed to merge the selectivity of an antigen-specific monoclonal antibody with an active cytotoxic payload via a unique hydrolyzable linker, thereby maximizing the therapeutic window of the drug. Among these agents, Sacituzumab Govitecan has demonstrated significant clinical benefit in patients with metastatic breast cancer, including those with triple-negative disease and endocrine-resistant ERα-positive tumors (mTBNC, ASCENT phase-3 clinical trial) (17, 18). Moreover, the TROPiCS-02 study also showed encouraging primary results in a heavily pretreated, endocrine-resistant ERα-positive breast cancer population (19). Nevertheless, despite the promising efficacy of ADCs, the emergence of drug resistance remains a major limitation to their clinical benefit. The mechanisms underlying resistance to ADCs are multifactorial and may involve several steps in the drug action process. Among these, antigen–antibody binding, loss of antigen expression, and changes in the tumor microenvironment appear to be critical determinants of ADC efficacy (18, 20).

Accumulating evidence indicates that TROP2 expression is dynamically modulated by microenvironmental cues, including fibroblast growth factor 10 (FGF10), tumor necrosis factor alpha (TNF-α), and transforming growth factor beta (TGF-β) (21–23). Lu et al. demonstrated that FGF10, a growth factor implicated in multiple cancers, induces TROP2 expression during lung bud morphogenesis (21, 24). In the context of colon cancer, low concentrations of TNF-α, a key inflammatory cytokine within the tumor microenvironment, have been shown to upregulate TROP2 expression through activation of the ERK1/2 signaling pathway, thereby promoting cancer cell migration and invasion (22). Moreover, TGF-β1, a pleiotropic cytokine involved in cancer development and progression, has been reported to upregulate TROP2 expression in human epidermal Langerhans cells (23).

Beyond malignant epithelial cells, TROP2 has also been detected in non-tumor components of the breast microenvironment, including mammary adipocytes (25), suggesting a potential bidirectional interaction between stromal elements and TROP2-expressing cells.

To date, the direct mechanisms through which adipocytes regulate TROP2 expression remain poorly understood. In this study, we investigated whether adipocyte-derived factors modulate TROP2 expression in breast cancer cells and explored how obesity-associated alterations in the adipose microenvironment may influence the responsiveness to TROP2-directed antibody–drug conjugates.

## 2 Materials and methods

### 2.1 Antibodies and reagents

Antibodies against TROP2 (ab227689) was acquired from Abcam (Cambridge, UK), while antibodies against β-actin (sc-69879) and GAPDH (sc-47724) from Santa Cruz Biotechnology (Dallas, TX, USA). (Z)-4-Hydroxytamoxifen (H7904) was acquired from Sigma-Aldrich (Milan, IT). ICI 182, 780 (ICI) was purchased from Zeneca Pharmaceuticals (Cheshire, UK). Sacituzumab Govitecan (SG, D4002), Datopotamab Deruxtecan (DD, D4050), and Anastrozole (S1188) were obtained from Selleck Chemicals (Houston, TX, USA). 17β-estradiol (3301) was obtained from Sigma-Aldrich.

### 2.2 Cell cultures

Human MCF-7 (HTB-22), T47-D (HTB-133), MDA-MB-231 (HTB-26) and 3T3-L1 mouse pre-adipocytes (CL-173) were purchased from the American Type Culture Collection (Manassas, VA, USA) and cultured following supplier’s instruction. MCF-7 and MDA-MB-231 cells were cultured in DMEM-F12 medium, containing 1% L-glutamine, and 1% penicillin-streptomycin and 10% or 5%FBS, respectively (Thermo Fisher Scientific, Waltham, MA, USA). T47-D cells were cultured in RPMI 1640 medium (Thermo Fisher Scientific), supplemented with 10% FBS and 1% penicillin-streptomycin. 3T3-L1 cells were cultured in DMEM High Glucose medium (Thermo Fisher Scientific) containing 10% NewBorn Calf Serum (Thermo Fisher Scientific) and 1% penicillin/streptomycic. All cell Lines were maintained at 37 °C in a humidified atmosphere containing 5% CO_2_ and were authenticated every 6 months at our Sequencing Core and regularly tested for mycoplasma-negativity (MycoAlert, Lonza, Basel, CH). The cell lines were used within 3 months of culture and before the 20^th^ passage.

### 2.3 3T3-L1 adipocyte differentiation

3T3-L1 mouse pre-adipocytes cell line were grown to 95–97% confluence in complete medium. After confluence (day 4), the medium was replaced by Differentiation Medium: DMEM High Glucose containing 0.5 mM isobutylmethylxanthine (IBMX; I5879, Sigma-Aldrich, Milan, IT), 1 µg/ml bovine insulin (I0516, Sigma-Aldrich, Milan, IT), and 1 µM dexamethasone (D4902, Sigma-Aldrich, Milan, IT) supplemented with 10% FBS and 1% P/S. After two days (day 6), the medium was changed to Basal Differentiation Medium: DMEM High Glucose, supplemented only with 10% FBS and 1 µg/ml bovine insulin. At the end of the differentiation process (day 21) adipocytes characterization was conducted on mature adipocytes (3T3-L1A) via phase-contrast microscopy to verify the morphology, and RNA extraction was performed for the purpose of analyzing the mRNA levels of specific adipocyte differentiation-related genes.

### 2.4 Conditioned medium systems

Mature adipocytes (3T3-L1A) were incubated in 3% Charcoal-stripped Fetal Bovine Serum (CS, Sigma-Aldrich) phenol-red free media for 24h. CM was collected, centrifuged, and used in respective experiments. In a separate set of experiments, 3T3-L1A were treated with Anastrozole for 24 h, and CM was collected and processed as described above.

### 2.5 Real-time RT-PCR assay

Total RNA from cells was extracted by using the TRIzol reagent (Thermo Fisher Scientific). A NanoDrop-1000 spectrophotometer was used to evaluate the RNA concentration and quality. Two micrograms of total RNA were reverse transcribed with the RETROscript Kit (Applied Biosystems, Monza, IT). Gene expression was analyzed by real-time RT-PCR using SYBR Green Universal PCR Master Mix (Bio-Rad, Hercules, CA, USA) in an iCycler iQ Detection System (Bio-Rad). Relative gene expression levels were assessed and calculated as previously described (26). Each sample was normalized on 18S mRNA expression for human genes and on 36B4 mRNA expression for mouse genes. The primers used are listed in **Table 1**.

**Table 1:** Oligonucleotide primers used in this study.

Gene Name	Gene Symbol	Species	Primer sequences
Adiponectin	AdipoQ	*Mouse*	Forward 5’-ATGGTCCTGTGATGCTTTGA-3’ Reverse 5’-GAGAGTAAATGCACCAATAAG-3’
Fatty Acid Binding Protein 4	*FABP4*	*Mouse*	Forward 5’-ATGAAATCACCGCAGACGAC-3’ Reverse 5’-TCATAACACATTCCACCACC-3’
Leptin	*Ob*	*Mouse*	Forward 5’-ACTTCATTCCTGGGCTTCAC-3’ Reverse 5’-GATTCTCCAGGTCATTGGCT-3’
Peroxisome Proliferator Activated Receptor Gamma	*PPARG*	Mouse	Forward 5’-GGCTTCATGACAAGGGAGTTTC-3’ Reverse 5’-AACTCAAACTTGGGCTCCATAAAG-3’
Tumor-Associated Calcium Signal Transducer 2	*TACSTD2*	*Human*	Forward 5’-GCCTTCAACCACTCAGACCT-3’ Reverse 5’-GAGACTCGCCCTTGATGTCC-3’
18s rRNA	*18s*	*Human*	Forward 5’-CGGCGACGACCCATTCGAAC-3’ Reverse 5’-GAATCGAACCCTGATTCCCCGTC-3’
Ribosomal Protein Lateral Stalk Subunit P0	*36B4*	*Mouse*	Forward 5’-AGATTCGGGATATGCTGTTGG -3’ Reverse 5’-AAAGCCTGGAAGAAGGAGGTC-3’

### 2.6 Immunoblot analysis

Cells were lysed in RIPA Buffer (50 mM Tris-HCl, 150 mM NaCl, 1% Nonidet P-40, 0.5% sodium deoxycholate, 2 mM sodium fluoride, 2 mM EDTA, and 0.1% SDS) containing a mixture of protease inhibitors (aprotinin, phenylmethylsulfonyl fluoride, and sodium orthovanadate). Equal amounts of cells extracts were resolved on 10% SDS-polyacrylamide gel as previously described (27). Odyssey FC (Licor, Lincoln, NE, USA) and Image Studio Lite software were employed to acquire and quantify the band of interest.

### 2.7 Enzyme-linked immunosorbent assay

Levels of estrogens were measured in the CM of adipocytes using mouse Estrogen ELISA kit according to manufacturer’s instructions (ab285291, Abcam). Specifically, 3T3-L1A cells were maintained in 3% Charcoal-stripped Fetal Bovine Serum (CS, Sigma-Aldrich) phenol-red free media for 24h and 10ml of supernatants were collected, centrifuged and lyophilized for 1 night. The lyophilized sample was resuspended in 200ul of DMEM only and used for analyses.

### 2.8 Expression profiling of TACSTD2 in a public breast cancer dataset stratified by BMI and ER status

TACSTD2 expression was in a public dataset, comparing normal weight (NW) and overweight/obese (OW/Ob) breast cancer patients stratified accordingly to Estrogen Receptor α (ER α) status, using a one-sided Mann-Whitney U test after back-transformation of log2 values (28).

### 2.9 Shortest path network analysis of nuclear estrogen receptor binding interactors

Interactors of the Gene Ontology Molecular Function Nuclear Estrogen Receptor Binding (GO:0030331) were functionally processed by applying the MetaCore™ integrated software suite for functional analysis of experimental data (Clarivate Analytics, London). By using the network building tool and its shortest path algorithm and allowing a maximum of two steps in connecting seed nodes, proteins encoded by the 61 genes associated with this GO term were functionally crosslinked to generate shortest path networks (SPNs). The SPNs were constructed by limiting protein processes to individual proteins, excluding their involvement in multimeric complexes, and avoiding canonical pathways. According to the manually curated MetaCore in-house database of physiological and pathological protein–protein and protein–nucleic acid interactions, signaling and metabolic pathways derived from the scientific literature, only proteins known to be closely and functionally related were included in the resulting network(s). The obtained SPNs provide an interactomic overview of the molecular framework underlying estrogen receptor binding and were prioritized according to their statistical significance (p ≤ 0.001). The most significant SPN was graphically visualized by nodes (proteins) and edges (functional interconnections) and named according to its five most significant nodes, identified as central hubs. To explore the potential involvement of TACSTD2 in estrogen receptor–related processes, TACSTD2 was subsequently introduced into the network as an additional object, allowing the assessment of its possible functional connections within the established interactomic framework.

### 2.10 MTT assays

Cell viability was determined by using 3-(4,5-dimethylthiazol-2-yl)-2,5 diphenyltetrazolium bromide (MTT, Sigma–Aldrich) reagent as previously described (6).

### 2.11 Kaplan-Meier survival analysis

Kaplan–Meier survival analysis was performed using the KM-plotter online tool (http://kmplot.com/analysis/), which integrates gene expression and survival data from publicly available breast cancer datasets. Patients were stratified into high and low expression groups using the auto-selected cutoff. Survival differences were assessed using the log-rank test, and hazard ratios (HRs) were calculated by the tool. A p-value < 0.05 was considered statistically significant.

### 2.12 Statistical analysis

Statistical analysis was performed using the Student’s t-test or one-way ANOVA, as appropriate, using GraphPad Prism 8 (GraphPad Software, Inc., San Diego, CA, USA). Data are presented as mean ± SEM from at least three independent experiments. A p value < 0.05 was considered statistically significant.

## 3 Results

### 3.1 Effects of adipocyte secretome on TROP2 expression in breast cancer cells

**Figure 1:**
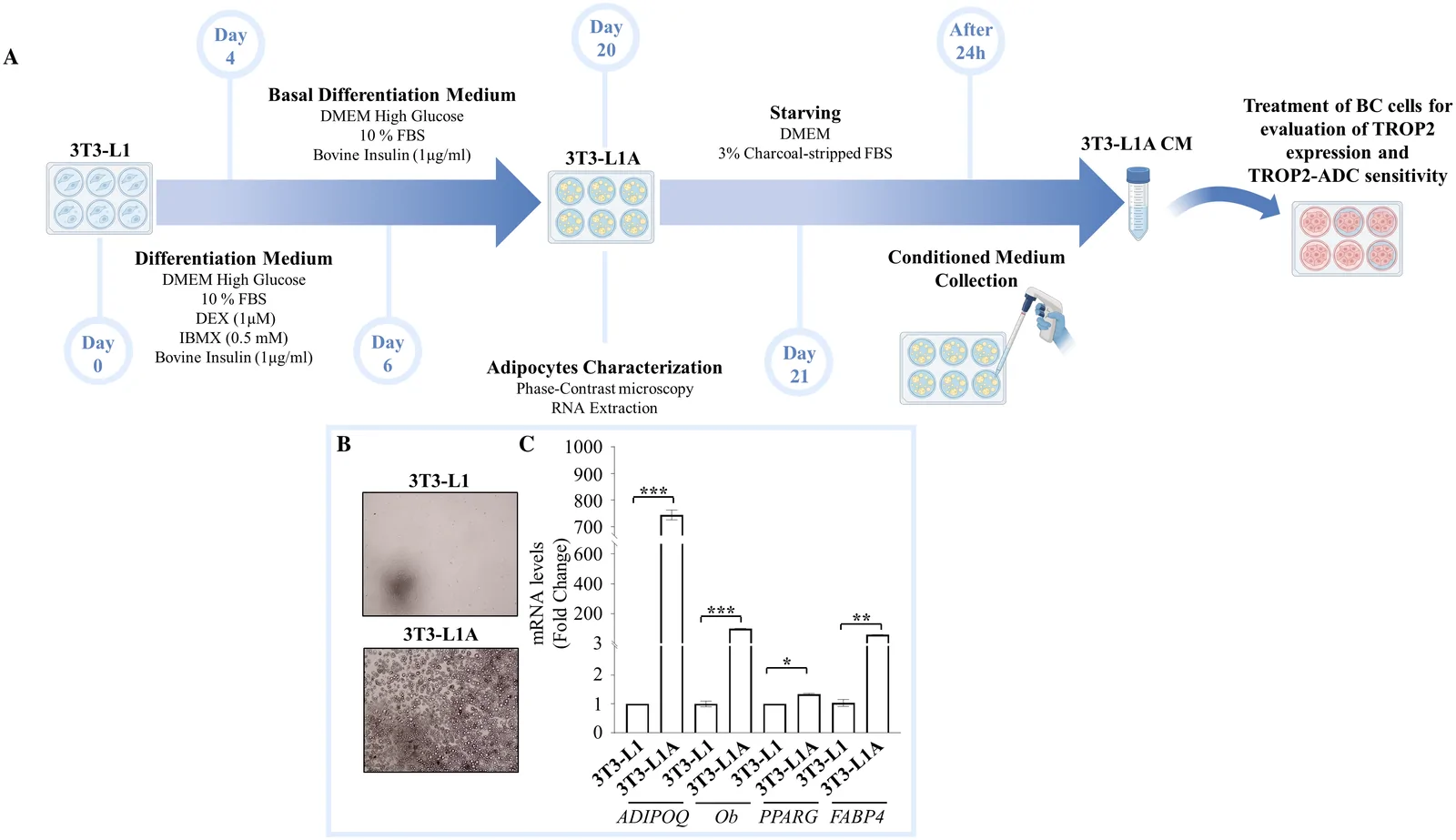
Experimental workflow and characterization of 3T3-L1 adipocyte differentiation. **(A)** Schematic timeline of 3T3-L1 differentiation into mature adipocytes (3T3-L1A) and overview of the experimental workflow used to assess changes in breast cancer (BC) cells induced by conditioned media (CM) derived from 3T3-L1A. **(B)** A typical bright-field picture of 3T3-L1 and 3T3-L1 A adipocytes characterized by accumulation of lipid droplets is shown (40X). **(C)** RT-PCR analysis for the mRNA levels of specific adipocyte differentiation related genes (Adiponectin, *ADIPOQ*, leptin, *Ob*, peroxisome proliferator-activated receptor gamma, *PPARG* and fatty acid binding protein 4, *FABP4*). Data are expressed as means ± S.E.M. of three different experiments, each performed in triplicate. *P < 0.05; **P < 0.005; ***P < 0.0005.

To carry out this project, we employed 3T3-L1 adipocytes, a well-established *in vitro* model that is widely used to investigate adipogenesis and adipocyte biology. Fully differentiated adipocytes were obtained by exposing 3T3-L1 pre-adipocytes to a differentiation medium, followed by maintenance in basal differentiation medium for at least 21 days, allowing the acquisition of a mature adipocyte phenotype (**Figure 1A**). Representative phase-contrast images showed the characteristic morphological changes associated with adipogenic differentiation, including increased cell size and progressive accumulation of intracellular lipid droplets in 3T3-L1 adipocytes (**Figure 1B**). The efficiency of the differentiation process was further validated by the relative quantification of adipocyte-specific markers. In particular, adiponectin (*ADIPOQ*), leptin (*Ob*), peroxisome proliferator-activated receptor gamma (*PPARG*), and fatty acid-binding protein 4 (*FABP4*) were significantly upregulated in differentiated adipocytes compared with pre-adipocytes (**Figure 1C**), confirming the successful differentiation of 3T3-L1 cells into mature adipocytes. Subsequently, to better recapitulate the tumor microenvironment and adipocyte–cancer cell crosstalk, *in vitro* co-culture systems were employed in order to evaluate TROP2 expression in the breast cancer cells together with the sensitivity to TROP2-targeting antibody–drug conjugates (TROP2-ADCs).

**Figure 2:**
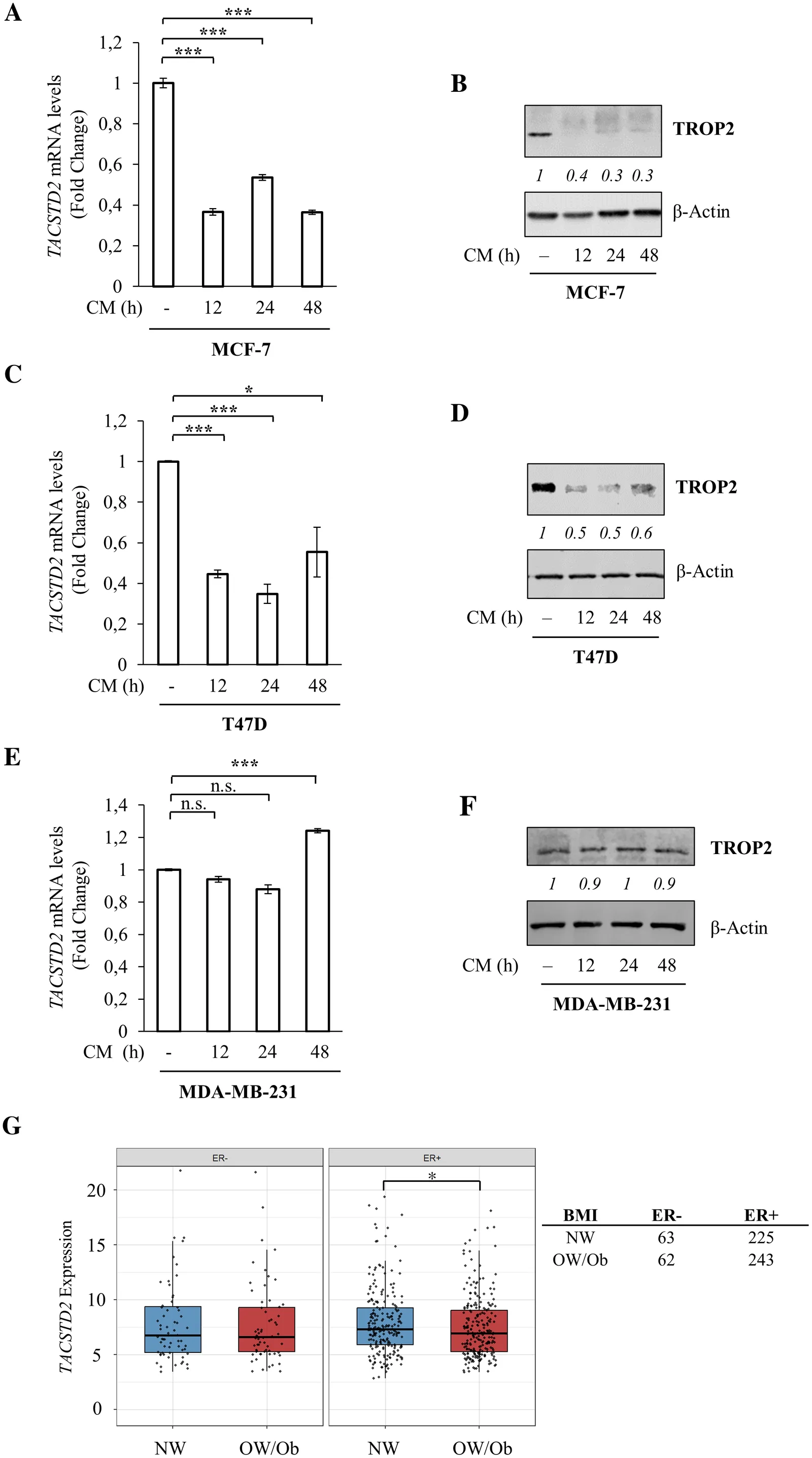
Effects of adipocyte-derived CM on TROP2 expression in BC cells. Realtime RT-PCR analysis for *TACSTD2* mRNA expression in MCF-7 **(A)**, T47D **(C)** and MDA-MB-231 **(E)** breast cancer cells treated with or without (-) 3T3-L1A-CM. Immunoblotting showing TROP2 expression in MCF-7 **(B)**, T47D **(D)** and MDA-MB-231 **(F)** breast cancer cells treated with or without (-) 3T3-L1A-CM. β-Actin was used as a control for equal loading and transfer. Numbers below the blot represent the mean of the band optical density of two separate experiments expressed as fold change over untreated (-) cells. **(G)** One-sided Mann-Whitney U test for evaluating *TACSTD2* expression in Normal Weight (NW, n = 288) and Overweight/Obese (OW/Ob, n = 305) breast cancer patients categorized in ERα-positive and ERα-negative groups. Data are expressed as means ± S.E.M. of three different experiments, each performed in triplicate. n.s., non-significant; *P < 0.05; ***P < 0.0005.

By analyzing our previous RNA sequencing data investigating the role of adipocyte secretome in modulating the transcriptome of MCF-7 breast cancer cells (29), we revealed a significant down-regulation of *TACSTD2*, the gene encoding the protein TROP2, in ERα-positive Luminal-like MCF-7 cells treated with conditioned media (CM) derived from 3T3-L1A (fold change = -2.52, padj = 2,32E-89). To validate this observation, MCF-7 breast cancer cells were incubated with 3T3L1A-CM and TROP2 mRNA and protein expression were assessed by qRTPCR, and immunoblotting. As shown in **Figure 2A**, we found significant reduced *TACSTD2* mRNA levels in MCF-7 cells upon exposure to 3T3-L1 A CM for 12, 24 and 48h. Concerning protein expression, the maximal reduction of TROP2 was also reached as early as 12h and was maintained throughout the 48h treatment period, as demonstrated by immunoblot analysis (**Figure 2B**). *TACSTD2* mRNA and TROP2 protein levels were also significantly down-regulated in ERα-positive Luminal-like T47D breast cancer cells exposed to 3T3-L1 A-derived CM (**Figures 2C, D**). Interestingly, no significant changes were observed in triple-negative MDA-MB-231 breast cancer cells at either the mRNA or protein level (**Figures 2E, F**), suggesting that the effects of adipocyte-derived CM on TROP2 expression are molecular subtype–dependent. Furthermore, TROP2 expression was analyzed in a public dataset, comparing normal weight (NW) and overweight/obese (OW/Ob) breast cancer patients to explore a potential association between *TACSTD2* expression and obesity. Remarkably, a significant reduction of *TACSTD2* expression in the OW/Ob group compared to NW breast cancer patients was found within the estrogen receptor (ER) α-positive subgroup (p = 0.026), whereas no significant differences were detected in ER α-negative breast cancer patients (**Figure 2G**).

### 3.2 Effects of estrogen receptor signaling activation on TROP2 expression

**Figure 3:**
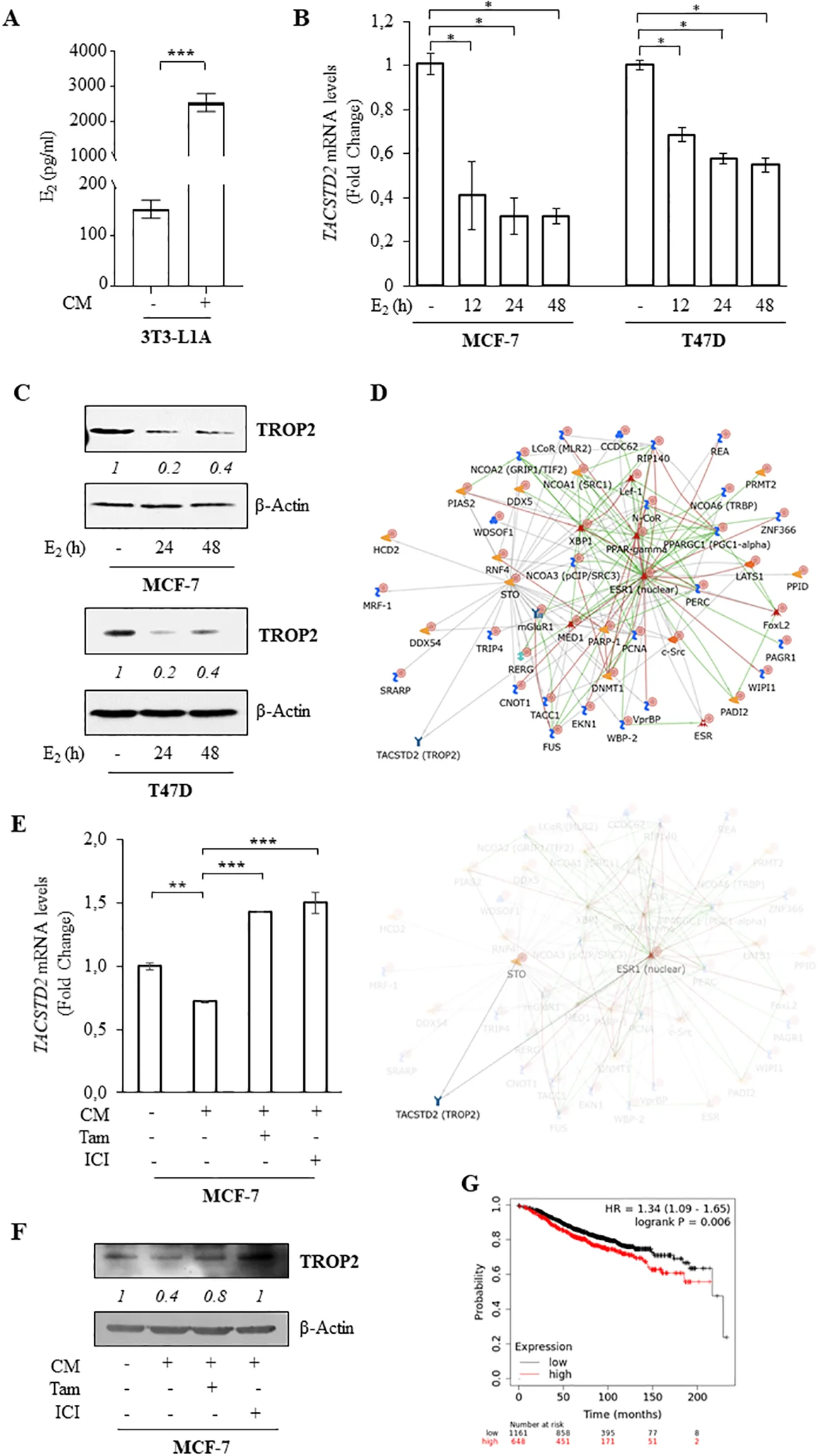
Impact of estrogen receptor pathway activation on TROP2 levels. **(A)** Enzyme linked immunosorbent assay (ELISA) to measure estrogen levels in CM derived from 3T3-L1A. **(B)** Realtime RT-PCR analysis for *TACSTD2* mRNA expression in MCF-7 and T47D breast cancer cells treated with or without (-) 17β-estradiol (E_2_, 100nM) for 12, 24 and 48h. **(C)** Immunoblotting showing TROP2 expression in MCF-7 and T47D breast cancer cells treated with or without (-) 17β-estradiol (E_2_, 100nM) for 24 and 48h. β-Actin was used as a control for equal loading and transfer. Numbers below the blot represent the mean of the band optical density of two separate experiments expressed as fold change over untreated (-) cells. **(D)** *Upper panel*: Direct Interaction Network (DIN) analysis of “nuclear estrogen receptor binding” ontology. The resulting network displayed a highly centralized topology; *lower panel*: *TACSTD2* was added as network object and showed direct interactions with the two main central hubs, including *ESR1*. **(E)** Realtime RT-PCR analysis for *TACSTD2* mRNA expression in MCF-7 breast cancer cells treated with or without (-) Tamoxifen (Tam, 1µM) and Fulvestrant (ICI, 1µM) for 48 hours. **(F)** Immunoblotting showing TROP2 expression in MCF-7 breast cancer cells treated with or without (-) Tamoxifen (Tam, 1µM) and Fulvestrant (ICI, 1µM) for 48 hours. β-Actin was used as a control for equal loading and transfer. Numbers below the blot represent the mean of the band optical density of two separate experiments expressed as fold change over untreated. **(G)** Kaplan–Meier survival analysis in breast carcinoma patients with high *TACSTD2* and low *ESR1* in luminal subgroups (*n* = 1809). Data are expressed as means ± S.E.M. of three different experiments, each performed in triplicate. *P < 0.05; **P < 0.005; ***P < 0.0005.

The subtype-specific effect observed exclusively in ERα-positive cells prompted us to explore the potential involvement of the estrogen signaling pathways. To this aim, we first quantified estrogen levels in the CM derived from adipocytes by ELISA assay. Notably, the estrogen concentrations were significantly higher in adipocyte-derived CM than in the basal medium (**Figure 3A**). Therefore, based on this observation, we next specifically evaluated the effects of estradiol (E_2_) exposure on TROP2 expression in breast cancer cells. Particularly, the concentration of 17β-estradiol used in our experiments was selected to model a condition of enhanced estrogenic stimulation that may occur within adipose-rich breast tumor microenvironments. This approach was intended to investigate whether robust ERα activation, potentially associated with increased local estrogen production resulting from enhanced aromatase activity in obesity-associated adipose tissue (3, 30–32), could modulate TROP2 expression. Thus, MCF-7 and T47-D cells were treated with E_2_ for 12, 24, and 48h. Notably, E_2_ treatment significantly reduced TROP2 expression at both the mRNA and protein levels (**Figures 3B, C**), suggesting that ERα signaling may be involved in the modulation of TROP2 expression.

In light of the potential estradiol-dependent regulation of TROP2 observed in ERα-positive cells, we next explored whether a possible functional interaction exists between ERα and TROP2. The “nuclear estrogen receptor binding” ontology was analyzed using MetaCore and Direct Interaction Network (DIN) analysis to identify key functional regulators involved in estrogen-dependent nuclear signaling. The resulting network displayed a highly centralized topology. As expected, *ESR1* emerged as one of the main hubs of the network, confirming the biological relevance of the selected ontology and underscoring the central role of estrogen receptor signaling in nuclear transcriptional regulation. In addition, Nuclear Receptor Binding SET Domain Protein 1 (*STO*) was identified as a second highly connected node, further supporting its involvement in the estrogen receptor–associated regulatory network (**Figure 3D**, *upper panel)*. Although *TACSTD2* was not originally included in the ontology, its external integration into the network revealed direct interactions with the main hubs, including *ESR1* and *STO*. This finding suggests that *TACSTD2* may be functionally linked to the estrogen receptor signaling machinery. The positioning of *TACSTD2* within the network indicates a potential role as a connector between upstream or non-nuclear signaling components and estrogen-dependent transcriptional regulation, despite *TACSTD2* not being a canonical nuclear estrogen receptor–binding protein (**Figure 3D**, *lower panel*).

To further assess whether ERα activity mediates the effects of adipocyte-derived CM on TROP2 expression, MCF-7 cells were treated with the selective estrogen receptor modulator tamoxifen (TAM) and the selective estrogen receptor degrader fulvestrant (ICI 182,780; ICI) in the presence of adipocyte-derived CM. Both compounds were able to counteract the downregulatory effects of adipocyte-derived CM on TROP2 mRNA and protein levels (**Figures 3E, F**). To confirm the contribution of adipocyte-derived estrogens, mature adipocytes were treated with the aromatase inhibitor Anastrozole (ANA) to inhibit estrogen production and the resulting CM was collected and applied to MCF-7 cells. Notably, CM derived from ANA-treated adipocytes failed to downregulate TROP2 expression at both the mRNA and protein levels (**Supplementary Figure 1**).

Together, these findings suggest that adipocyte-induced downregulation of TROP2 may depend on ERα signaling, without establishing a direct transcriptional control of TACSTD2 by ERα.

Finally, the clinical significance of ERα and TROP2 in human breast cancers have been evaluated by Kaplan-Meier analysis. Patients with high *TACSTD2*/low *ESR1* expression exhibited a slightly worse survival in the luminal subgroup (**Figure 3G**). These findings are consistent with a potential inverse relationship between *TACSTD2* and *ESR1* expression.

### 3.3 Effects of adipocyte-derived conditioned media on the efficacy of TROP2-directed antibody–drug conjugates

**Figure 4:**
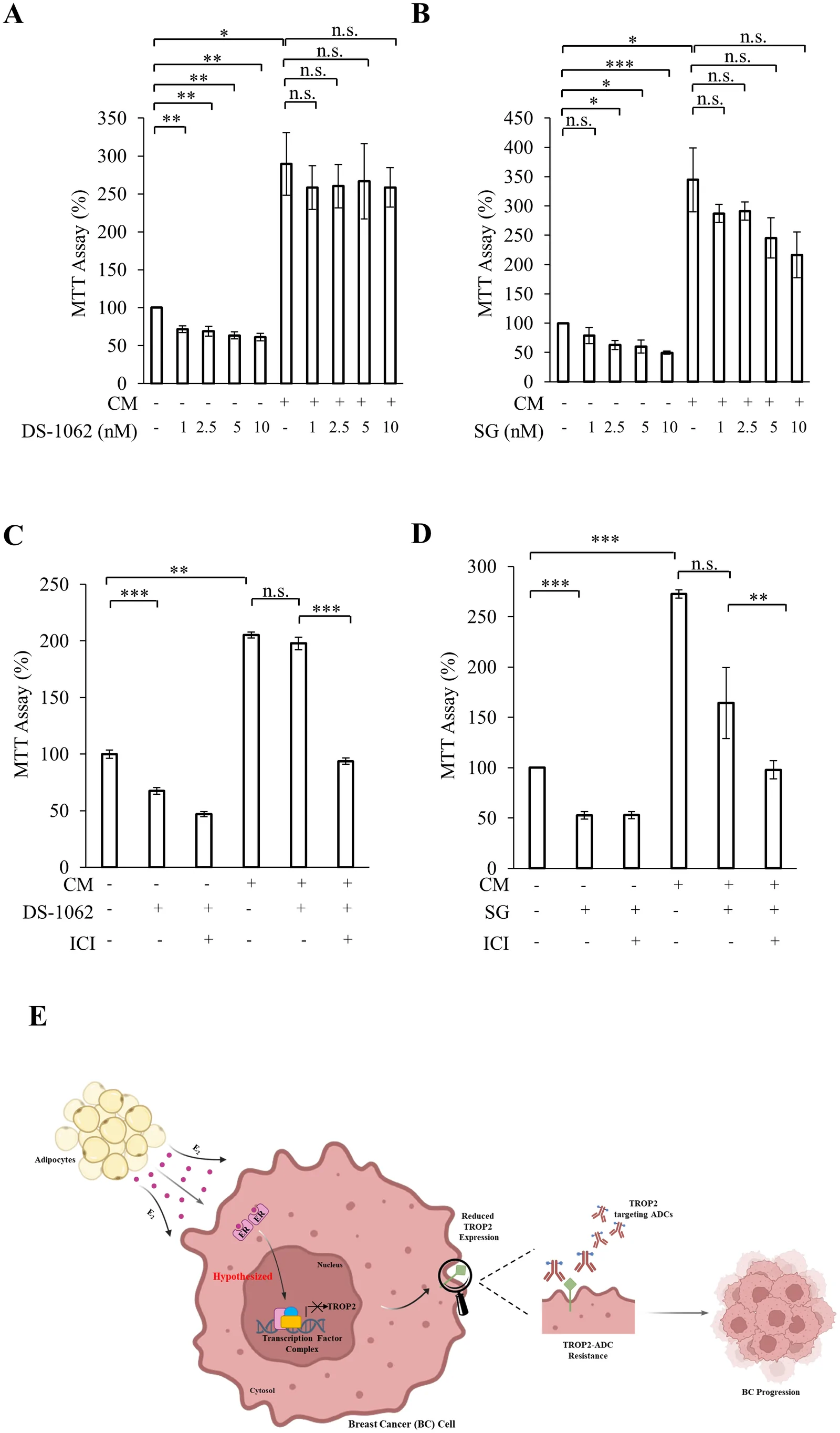
Adipocyte-derived conditioned medium reduced sensitivity to TROP2-ADCs in ERα-positive MCF-7 breast cancer cells. MTT Assays in MCF-7 breast cancer cells treated with or without (-) CM isolated from mature adipocytes (3T3-L1 A CM) in the presence or not of TROP2-ADCs, Datopotamab Deruxtecan (DS-1062, **(A)**) and Sacituzumab Govitecan (SG, **(B)**) for 72 hours. MTT Assays in MCF-7 breast cancer cells treated with CM isolated from mature adipocytes (3T3-L1 A CM) in the presence or not of TROP2-ADCs, Datopotamab Deruxtecan (DS-1062, 5nM, **(C)**) and Sacituzumab Govitecan (SG, 5nM, **(D)**) and Fulvestrant (ICI, 1µM) for 72 hours. Data are expressed as means ± S.E.M. of three different experiments, each performed in triplicate. n.s. non-significant; *P < 0.05; **P < 0.005; ***P < 0.0005. **(E)** Schematic illustration of adipocytes-breast cancer (BC) cell crosstalk. Adipocytes release 17β-estradiol (E_2_), which acts in a paracrine manner on ERα-positive BC cells by binding estrogen receptor α (ERα). Upon activation, ERα signaling is proposed to reduce TACSTD2 expression through ERα-dependent regulatory mechanisms that remain to be fully elucidated and may involve indirect transcriptional pathways. The resulting decrease in TROP2 levels may attenuate the sensitivity of BC cells to TROP2-targeting antibody–drug conjugates (ADCs), potentially promoting tumor cell progression.

Given that loss or reduction of TROP2 is a known mechanism of resistance to TROP2-targeting therapies (33, 34), we investigated whether the adipocyte secretome, by down-regulating TROP2 expression, could reduce the sensitivity of MCF-7 breast cancer cells to TROP2-directed antibody–drug conjugates (ADCs), including Datopotamab Deruxtecan (DS-1062) and Sacituzumab Govitecan (SG). Thus, MCF-7 cells were treated with DS-1062 per 72h in the presence or absence of 3T3L1A-derived CM. As shown in **Figure 4A**, DS-1062 significantly decreased cell viability starting at 1 nM, with a maximal reduction of approximately 40% at 10 nM. In contrast, when cells were exposed to 3T3-L1A CM, DS-1062 did not significantly affect cell viability. Similarly, SG treatment reduced cell viability at concentrations ranging from 2.5 to 10 nM, reaching a maximum growth inhibition of approximately 50% at 10 nM. However, in the presence of 3T3-L1A CM, SG failed to significantly impact viability at any dose (**Figure 4B**).

To further investigate whether ERα signaling contributes to the reduced responsiveness to TROP2-directed ADCs induced by adipocyte-CM, MCF-7 cells were treated with ICI in combination with either SG or DS-1062. We found that pharmacological inhibition of ERα signaling significantly restored the antiproliferative activity of both TROP2-directed ADCs in the presence of adipocyte-CM (**Figures 4C, D**), providing functional evidence linking adipocyte-associated endocrine signaling, TROP2 regulation, and ADC response. Collectively, these findings suggest that the adipocyte secretome may impair the responsiveness of breast cancer cells to TROP2-targeting ADCs, likely through the activation of ERα-dependent signaling pathways.

We therefore propose a working model in which adipocyte-derived estrogen signaling contributes to TROP2 downregulation through ERα-dependent mechanisms. The resulting decrease in TROP2 levels may reduce the sensitivity of ERα-positive breast cancer cells to TROP2-directed ADCs, suggesting a potential link between adipocyte-driven estrogen signaling and reduced therapeutic responsiveness. Although our data support a functional involvement of ERα in this regulatory process, they do not establish direct transcriptional control of *TACSTD2* by ERα. Indeed, *TACSTD2* is not currently recognized as a canonical estrogen-responsive gene, and indirect mechanisms involving ERα-regulated gene networks or lineage-specific transcription factors may contribute to the observed effects. Further mechanistic studies will be required to elucidate this regulatory axis. The proposed hypothesis is summarized in the working model presented in **Figure 4E**.

## 4 Discussion

Adipocytes constitute the predominant cellular component of the mammary stroma and actively contribute to breast cancer biology beyond providing structural support (35, 36). In the present study, we identify a previously underexplored regulatory axis through which adipocytes influence the responsiveness of luminal ERα-positive breast cancer cells to TROP2-directed antibody–drug conjugates (ADCs) via paracrine estrogen pathway. Although adipocyte-tumor crosstalk is increasingly recognized as a determinant of therapy resistance, including chemotherapy, targeted therapy, hormonal therapy, and immune therapy (36), its impact on the efficacy of antibody–drug conjugates (ADCs) have not been systematically investigated.

Current evidence indicates that resistance to ADCs may be driven by microenvironmental factors as well as by adaptive cellular reprogramming induced by therapeutic pressure (37–40). Consistent with these observations, our findings reveal, for the first time, that adipocyte-derived conditioned media (CM) markedly counteracted the inhibitory effects of both TROP2-directed ADCs Datopotamab Deruxtecan and Sacituzumab Govitecan in ERα-positive breast cancer cells. Several mechanisms have been proposed to account for reduced responsiveness to ADCs, including downregulation of the target antigen, impaired ADC internalization, alterations in intracellular trafficking and payload release, and activation of pro-survival signaling pathways (33, 34). Among these, decreased expression or complete loss of the target antigen is particularly relevant, as it may compromise ADC internalization and payload delivery, ultimately attenuating antitumor activity. Here, we demonstrate that adipocyte-derived CM decreased TROP2 expression at both transcriptomic and proteomic levels in ERα-positive breast cancer cells, whereas no significant modulation was observed in ERα-negative models, supporting a cell type–specific effect. The concomitant reduction in TROP2 expression and responsiveness to TROP2-directed ADCs suggests that TROP2 downregulation may contribute to the observed phenotype. However, given the multifactorial nature of ADC response and the ability of ERα signaling to modulate cellular processes independently of TROP2 expression, additional mechanisms influenced by adipocyte-derived factors and/or ERα signaling cannot be excluded and warrant further investigation.

Breast adipose tissue functions as an active endocrine organ that secretes a wide array of bioactive molecules, including adipokines, cytokines, chemokines, growth factors, steroid hormones and extracellular vesicles that are critically involved in breast cancer development and progression (2–4, 6, 41–51). Among these signals, estrogens may be particularly relevant in ERα-positive breast cancer. Notably, Laforest et al. reported higher intratumoral estrogen levels in the adipose tissue of patients with ERα-positive breast cancer compared with ERα-negative cases (52). Indeed, substantial evidence supports the concept that mammary adipose tissue, through aromatase activity in adipose stromal cells, represents a relevant source of local estrogens that can sustain hormone-dependent breast cancer growth (3, 4, 53, 54). Consistent with this notion, we found that adipocytes secrete elevated levels of estrogen. Importantly, exposure of ERα-positive breast cancer cells to estradiol led to a significant reduction in TROP2 expression. Accordingly, CM obtained from adipocytes treated with the aromatase inhibitor Anastrozole failed to downregulate TROP2 expression. Furthermore, pharmacological inhibition of ERα counteracted CM-induced TROP2 downregulation, with fulvestrant also restoring the antiproliferative activity of both TROP2-directed ADCs. Nevertheless, the adipocyte secretome comprises multiple bioactive factors, several of which may interact with or converge on ERα signaling. Thus, while additional adipocyte-derived signals cannot be excluded, our findings support a relevant role for the adipocyte-derived estrogen/ERα axis in TROP2 regulation and ADC responsiveness. Interestingly, *in silico* analyses suggest a potential functional interaction between ERα and TROP2. Nevertheless, the molecular mechanisms underlying this association remain to be fully elucidated, and further studies will be required to determine whether *TACSTD2* represents a direct ERα target gene or whether the observed effects are mediated through indirect transcriptional mechanisms involving ERα-regulated gene networks or lineage-specific transcription factors.

A limitation of the present study is the use of murine 3T3-L1 adipocytes. Although this ‘*in vitro*’ model is widely used and well established for investigating adipocyte biology and adipocyte–tumor cell crosstalk, validation in primary human adipocytes and/or clinically relevant ‘ex vivo’ systems will be important to further confirm the physiological and translational relevance of our findings within the human breast tumor microenvironment. Despite this limitation, we observed a significant downregulation of TROP2 expression in a cohort of overweight/obese patients with ERα-positive breast cancer compared with normal-weight counterparts, providing additional clinical evidence linking adipose tissue dysfunction to TROP2 regulation. Notably, no significant association between elevated BMI and TROP2 expression was detected in the ERα-negative cohort, suggesting that adipocyte-driven modulation of TROP2 is restricted to a specific molecular subtype.

Collectively, our findings support a model in which adipocyte-derived estrogen signaling contributes to the modulation of TROP2 expression and may influence responsiveness to TROP2-directed ADCs in ERα-positive breast cancer. These data provide a rationale for future studies to determine whether obesity-associated metabolic and adipose tissue alterations may serve as predictive biomarkers of response to ADC-based therapies and whether ERα blockade could mitigate microenvironment-driven resistance mechanisms.

## Data Availability

The datasets presented in this study can be found in online repositories. The names of the repository/repositories and accession number(s) can be found below: https://www.ncbi.nlm.nih.gov/geo/query/acc.cgi?acc=GSE223853, GSE223853.
